# HIV-1 Genetic Diversity and Its Impact on Baseline CD4+T Cells and Viral Loads among Recently Infected Men Who Have Sex with Men in Shanghai, China

**DOI:** 10.1371/journal.pone.0129559

**Published:** 2015-06-29

**Authors:** Xiaoyan Li, Yile Xue, Hua Cheng, Yi Lin, Leiming Zhou, Zhen Ning, Xuqin Wang, Xiaolei Yu, Wei Zhang, Fangwei Shen, Xiaohong Zheng, Jing Gai, Xiaoshan Li, Laiyi Kang, Phillipe Nyambi, Ying Wang, Minghua Zhuang, Qichao Pan, Xun Zhuang, Ping Zhong

**Affiliations:** 1 Department AIDS and STD, Shanghai Municipal Center for Disease Control and Prevention, Shanghai Municipal Institutes for Preventive Medicine, Shanghai, China; 2 Public Health College, Nantong University, Nantong, Jiangsu Province, China; 3 Department of Pathology, New York University School of Medicine, New York, United States of America; Fudan University, CHINA

## Abstract

The HIV-1 epidemic among men who have sex with men (MSM) has been spreading throughout China. Shanghai, a central gathering place for MSM, is facing a continuously increasing incidence of HIV-1 infection. In order to better understand the dynamics of HIV-1 diversity and its influence on patient’s immune status at baseline on diagnosis, 1265 newly HIV-1-infected MSM collected from January 2009 to December 2013 in Shanghai were retrospectively analyzed for genetic subtyping, CD4+T cell counts, and viral loads. HIV-1 phylogenetic analysis revealed a broad viral diversity including CRF01_AE (62.13%), CRF07_BC (24.51%), subtype B (8.06%), CRF55_01B (3.24%), CER67_01B (0.95%), CRF68_01B (0.4%), CRF08_BC (0.08%) and CRF59_01B (0.08%). Twenty-four unique recombination forms (URFs) (1.98%) were identified as well. Bayesian inference analysis indicated that the introduction of CRF01_AE strain (1997) was earlier than CRF07_BC strain (2001) into MSM population in Shanghai based on the time of the most recent common ancestor (tMRCA). Three epidemic clusters and five sub-clusters were found in CRF01_AE. Significantly lower CD4+T cell count was found in individuals infected with CRF01_AE than in those infected with CRF07_BC infection (*P*<0.01), whereas viral load was significantly higher those infected with CRF01_AE than with CRF07_BC (*P*<0.01). In addition, the patients with >45 years of age were found to have lower CD4+T cell counts and higher viral loads than the patients with <25 years of age (*P*<0.05). This study reveals the presence of HIV-1 subtype diversity in Shanghai and its remarkable influence on clinical outcome. A real-time surveillance of HIV-1 viral diversity and phylodynamics of epidemic cluster, patient’s baseline CD4+T cell count and viral load would be of great value to monitoring of disease progression, intervention for transmission, improvement of antiretroviral therapy strategy and design of vaccines.

## Introduction

China at present is facing a new challenge for curbing the rapid spread of the HIV-1 epidemic through sexual transmission. According to an official report, since the first AIDS case was reported in 1985, about 154,000 patients died and people living with HIV/AIDS have reached 497,000 by the end of 2014. 104,000 new infections were diagnosed in calendar year 2014, with 14.8% increase over the previous year [1]. Significantly, of the new infections recorded in 2014, sexual transmission accounted for 91.5%, including 66% of heterosexual and 25% of homosexual cases (MSM). The fast increasing proportion for MSM transmission with 2.5% (2006), 13.7% (2011), and 25% (2014) in the HIV/AIDS infections has clearly shown that MSM has become a major risk factor in China. Recently, a large-scale cross-sectional survey on a national scale revealed a 4.9% of overall prevalence of HIV infection among MSM population [2].

Since the first HIV-1 case was identified in 1987 in Shanghai, the cumulative number of reported HIV-1-infected persons has reached close to 13,000 in 2014. HIV infection through sexual transmission accounted for 95%, including 60.98% and 34% for homosexual and heterosexual transmissions, respectively. It is estimated that approximate 8%-10% of MSM individuals were identified as HIV-1 infection among MSM population residing in Shanghai whereby the prevalence of HIV infection in this population rose from 1.5% in 2004 [3] to 6.8% in 2009 [2], and 7.5%-8.0% in 2011–2014 with an incidence of around 4.6% (unpublished data).

Nationwide molecular epidemiologic surveys and other studies indicated that CFR01_AE strain, initially prevailing in the heterosexual risk individuals in southwest border provinces and eastern coastal areas [4, 5], had quickly overtaken subtype B among MSM population over past few years [6–8]. Besides, CRF07_BC strain, the other dominating subtype among the injection drug user (IDU) risk group [9–11], was also reported to be spreading among MSM population in some provinces [8, 12–14]. Surprisingly, CRF55_01B strain, a newly identified recombinant virus, has already experienced an outbreak and been rapidly disseminated among MSM population and heterosexual individuals in Shenzhen city nearby HongKong years ago and elsewhere [15, 16]. Therefore, an ever-increasing migrant population and a sharply rising trend of HIV-1 infection in the MSM population in Shanghai would inevitably result in broadening and/or shifting the HIV-1 subtype diversity. Based on our previous studies [17] together with those of others [18–21] that revealed that some recently HIV-1-infected individuals rapidly experienced an outcome of AIDS after diagnosis and that some HIV-1 subtype infections were closely associated with disease progression, we hereby in this study expanded the study subjects covering all ages to investigate relatedness between the ongoing prevalent HIV-1 subtype strains and patients’ baseline-immune-status among MSM in Shanghai.

## Methods

### Study subjects

To analyze the HIV-1 genetic diversity, viral dynamics, and baseline immune-status among MSM population in recent five years (2009–2013) in Shanghai, we retrospectively investigated more than 2,000 HIV-1-infected MSM patient samples and their related clinical and demographic data, from which the sequences were isolated and analyzed based on our sentinel surveillance and drug resistance database.

To be included in the analysis, each case must 1) be diagnosed (Western blot confirmation) yearly for HIV-1 infection, 2) be collected from a MSM residing in Shanghai in recent 3 months, 3) be HAART-naïve before being sequenced, 4) have baseline CD4+ T cell count within 3 months after infection confirmation and continue to live in Shanghai for at least 6 months, and 5) have a high-quality sequence of at least 1000 nucleotides based on *pol* region.

Three criteria for determination of HIV-1 primary or recent infection have been extensively applied. They are defined by 1) diagnosis during clinically defined acute HIV infection (http://www.shcs.ch/56-definitions2.1) or 2) during recent infection defined by seroconversion (<1 year between last negative and first positive HIV test) or 3) by an ambiguous nucleotide count of <0.5% (in the first year) in a baseline in bulk sequencing of HIV-1 *pol* gene, from ART-naïve genetic sample. However, the surveillance analysis for acute and/or early infection of HIV-1 could only be restricted to low-density samples. Therefore, in order to exclude the all potential un-recent or late-chronic infections in our study, we chose the molecular analysis algorithm which might distinguish recent infection from long-standing infection (predictive value 98.7%) [22–24].

The study was reviewed and approved by the Institutional Review Board at the Human Medical Research Ethics Committee of the Shanghai Municipal Center for Disease Control and Prevention.

### CD4+ Lymphocyte count and viral load assay

Blood samples were collected within 3 months after infection had been confirmed. Absolute CD4+T lymphocytes were counted by flow cytometry (FACS Calibur, BD Company, USA) within 24 h after blood sample processing [17]. Plasma HIV-1 RNA viral load (part of samples) was quantified using CobasTaqMan System (Roche Diagnostics) according to the manufacture’s recommendation.

Our laboratory participates in the External Quality Assessment (EQA) for both CD4+T cell count and viral load assays, organized by National Reference Laboratory (NRL), China Center for Disease Control and Prevention (China CDC) and Australia NRL.

### Phylogenetic subtyping and evolutionary analysis

The preparation of HIV-1 genome RNA, RT-PCR amplification and DNA sequencing were performed as described in our previous study [17]. Multiple alignments were made using Gene Cutter (http://www.hiv.lanl.gov/content/index), with the selected reference sequences of various subtypes/recombinants from the Los Alamos HIV-1 database. A phylogenetic tree was constructed by the neighbor-joining method implemented by MEGA version 6.0 with the Kimura two-parameter and 1000 replicates for bootstrap analysis. New inter-subtype or inter-CRF sequences were analyzed by the recombination identification programs (RIP http://www.hiv.lanl.gov, and SimPlot 3.5.1 software).

Selection of the clustering sequences was analyzed by Maximum likelihood phylogeny with software Fasttree v2.1.7. Local support values were computed with the Shimodaira-Hassegawa test. The node SH-like support value of a cluster over 0.9 was considered credible.

To estimate the time of the most recent common ancestor (tMRCA) and the evolutionary rates for both CRF01_AE and CRF07_BC strains circulating in Shanghai, we selected the sequences close to node of each single epidemic cluster in Fasttree ML tree and performed Bayesian inference analysis using the Markov Chain Monte Carlo (MCMC) approach with chain length 200 million in BEAST v1.6.2. An HKY substitution model and uncorrelated relaxed log-normal molecular clock model and Bayesian skyline model were selected for the best-fit model for this analysis. The first 25% of states of each run were discarded as burn in. The convergence of parameters was checked using Tracer v1.5. The Maximum Clade Credibility (MCC) tree was constructed by TreeAnnotator v1.7.2 and shown by FigTree v1.3.1. The effective sample size (ESS) was above 200.

### Statistical analysis

Demographic characteristics were analyzed using chi-squared statistics (χ^2^ test or fisher exact test). Data of age-associated and genetic distances were described by the mean±standard deviation. The trend χ^2^ test was used to assess if there was a change in the trend of the proportions of subtypes from 2009 to 2013. Data of CD4 cell counts and viral load were describe by the median (Interquartile range IQR). Comparison of CD4+T cell counts and viral load between different subtypes was analyzed by Nonparameter Kruskal-Wallis H test, followed by a pairwise comparison with Bonferroni method. All tests were two-tailed and P values less than 0.05 were considered as statistically significant. All data were analyzed using SPSS20.0 software package (IBM company, New York, USA).

## Results

### Demographic characteristics of studied subjects

The demographic characteristics of these studied subjects were analyzed based on HIV-1 genetic subtypes. A total of 1,265 HIV-1-infected MSM individuals residing in Shanghai from 2009 to 2013 were enrolled according to the all patient/sample’s inclusion criteria of our study. The patients studied were of ages ranging from 18 to 79 years with age of 30.60±9.55 years old. Only 32.6% of these studied individuals were born in Shanghai, while 67.3% were migrants from other provinces, of whom 98% were of the Han ethnicity and 74.7% were single. Among the subjects with different levels of education, 49% had college training. However these HIV-1-infected individuals were unimaginably involved in various different occupations in different proportions, in which students accounted for 6.14% (see Table 1).

**Table 1:** Demographic characteristics of MSM newly infected by HIV-1 in Shanghai based on different subtypes.

	HIV-1 Subtypes						
	CRF01_AE(n = 786)	CRF07_BC(n = 310)	Subtype B(n = 102)	Others#(n = 67)	Total(n = 1265)	χ^2^	P value
**Sex and risk group** Male (MSM)	786(62.13)	310(24.51)	102(8.06)	67(5.30)	1265(100.00)		
**Years of diagnosis**						44.451	<0.001
2009	83(65.35)	30(23.62)	14(11.02)	0(0.00)	127(10.04)		
2010	149(74.50)	31(15.50)	17(8.50)	3(1.50)	200(15.81)		
2011	126(60.87)	58(28.02)	17(8.21)	6(2.90)	207(16.36)		
2012	162(56.64)	74(25.87)	22(7.69)	28(9.79)	286(22.61)		
2013	266(59.78)	117(26.29)	32(7.19)	30(6.74)	445(35.18)		
**Ages**						15.633	0.075
≤25	257(60.61)	112(26.42)	30(7.08)	25(5.90)	424(33.52)		
26–35	347(61.63)	146 (25.93)	42(7.46)	28(4.97)	563 (44.51)		
36–45	118 (68.21)	29 (16.76)	21(12.14)	5(2.89)	173(13.68)		
≥46	64(60.95)	23(21.90)	9 (8.57)	9(8.57)	105 (8.30)		
**Places of birth**						7.375	0.354
Shanghai	184(34.39)	61(27.11)	25(33.33)	17(37.78)	287(32.61)		
Other provinces	350(65.42)	164(72.89)	50(66.67)	28(62.22)	592(67.27)		
Unknown	1(0.18)	0(0.00)	0(0.00)	0(0.00)	1(0.11)		
**Nationality**						4.781	0.134
Han	530(99.06)	219(97.33)	73(97.33)	44(97.78)	866(98.41)		
Minority group	5(0.93)	6(2.67)	2(2.67)	1(2.22)	14(1.59)		
**Marriage Status**						7.044	0.613
Single	397(74.21)	165(73.33)	59(78.67)	36(80.00)	657(74.66)		
Married	56(10.47)	29(12.89)	9(12.00)	7(15.56)	101(11.48)		
Divorced/Widowed	57(10.65)	23(10.22)	4(5.33)	2(4.44)	86(9.77)		
Unknown	25(4.67)	8(3.556)	3(4.00)	0(0.00)	36(4.09)		
**Education**						7.415	0.234
Primary education	11(2.06)	1(0.52)	2(2.67)	0(0.00)	14(1.65)		
Secondary education	271(50.65)	98(50.52)	28(37.33)	22(48.89)	419(49.35)		
Higher education	253(47.29)	95(48.97)	45(60.00)	23(51.11)	416(49.00)		
**Occupation**						12.254	0.425
Students	32(5.98)	13(5.78)	7(9.33)	2(4.44)	54(6.14)		
Workers and farmersCivil servants *,	78(14.58)118(22.06)	22(9.78)60(26.67)	4(5.33)17(22.67)	7(15.56)14(31.11)	111(12.61)209(23.75)		
Service industry**	125(23.36)	53(23.56)	17(22.67)	8(17.78)	203(23.07)		
Others***	182(34.02)	77(34.22)	30(40.00)	14(31.11)	303(34.43)		
**Drug resistance**PDR^φ^PINRTINNRTI	30(3.82)14(1.78)12(1.53)11(1.40)	4 (1.29)1(0.32)2(0.65)1 (0.32)	2 (1.96)2 (1.96)1 (0.98)1 (0.98)	1(1.49)1(1.49)0(0.00)0(0.00)	37(2.92)18(1.42)11(0.87)11(0.87)	2.803	0.985

^#^ HIV-1 CRF55_01B, CRF59_01B, CRF67_01B, CRF68_01B and URFs.

* Carders and staffs serving for governments, enterprises, school and hospital

** People serving for commercial, catering and hair salon.

*** The unemployed, retirees and unclear.

^φ^ Primary drug resistance.

### HIV-1 CRF01_AE dominating MSM epidemic in Shanghai

**Fig 1:**
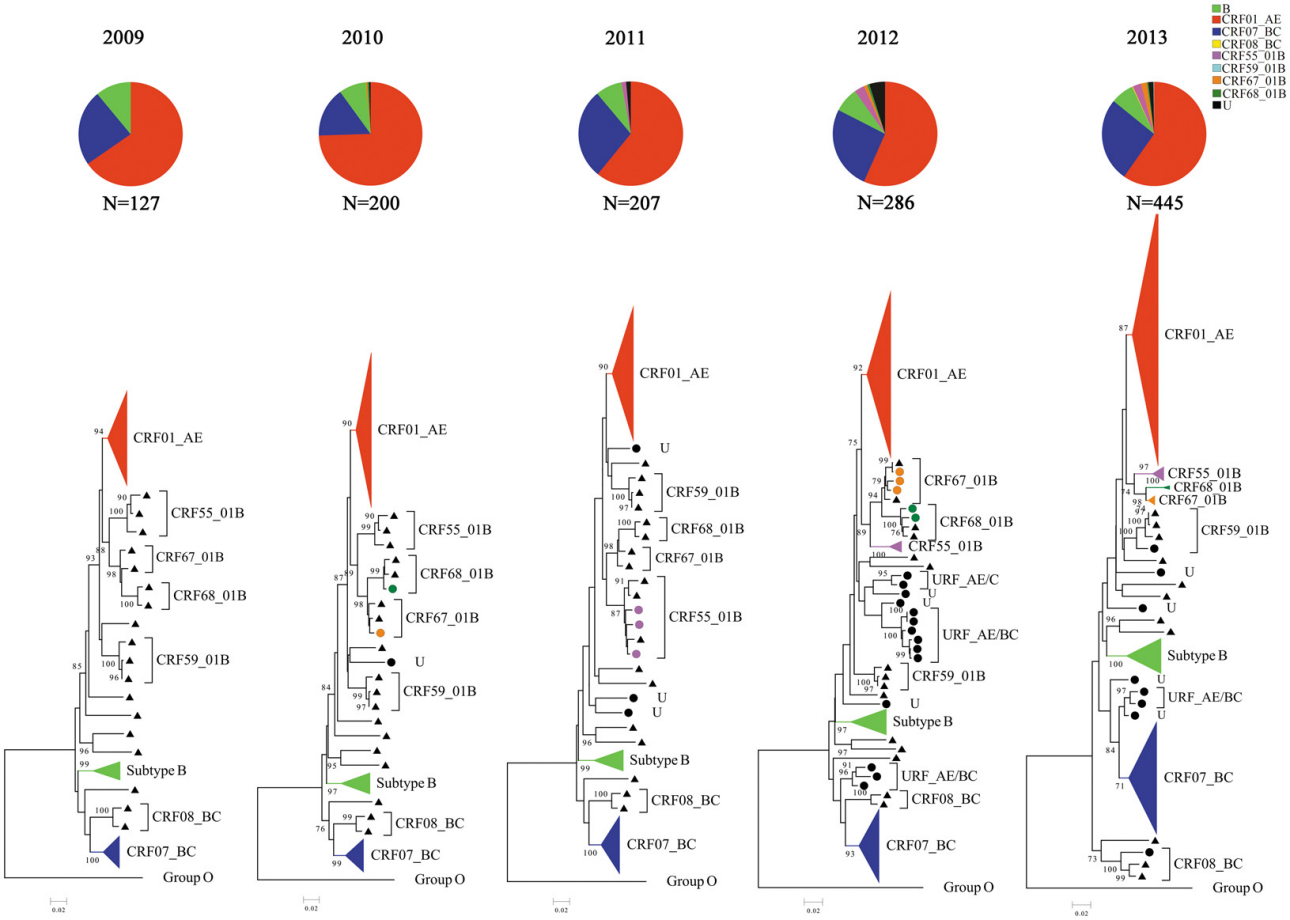
Phylogenetic tree analysis based on HIV-1 *pol* region among MSM who were newly infected from 2009 to 2013 in Shanghai. The phylogenetic trees were constructed using the Neighbor-Joining method. The bootstrap values of 1000 replicates above 70% are marked on the cluster nodes. CRF01_AE sequence cluster is shown as a triangle in red, CRF07_BC cluster in blue, subtype B cluster in green, and CRF55_01B cluster in pink. U, ambiguous/unidentified subtypes or recombinants and is marked as a solid circle in black. The reference sequences obtained from the Los Alamos HIV database are marked as a solid triangle in black. Trees were rooted by group O. The pie figures presented the proportion of analyzed subtypes represented in the corresponding phylogenetic trees.

Phylogenetic subtyping analysis showed the existence of viral diversity of HIV-1 in Shanghai where CRF01_AE was the most predominantly circulating strain, revealing the highest prevalence during the study period from 2009 to 2013 (62.13%, n = 786), followed by CRF07_BC (24.51%, n = 310), subtype B (8.06%, n = 102), CRF55_01B (1.9%, n = 24), CRF67_01B (0.95%, n = 12), and CRF68_01B (0.40%, n = 5). However, only one for each CRF08_BC and CRF59_01B strain was found in this study (0.08%), (Fig 1). Besides, by observation of the phylogenetic analysis based on the *pol* gene for CRF01_AE, CRF07_BC and subtype B strains during past five years, our study presented a different epidemic change, whereby only the CRF01_AE was decreasing (χ^2^ = 18.416, P = 0.001; Trend: χ^2^ = 8.713. P-trend = 0.003). It is worth noting that CRF55_01B, a newly identified CRF strain in China [15, 16], was firstly detected in calendar year 2011 and the proportion of this new viral strain has been increasing during the last three years (χ^2^ = 10.931, P = 0.018; Trend: χ^2^ = 8.468, P-trend = 0.004), while the other two newly found CRF67_01B and CRF68_01B strains [12] already existed in year 2010 in Shanghai. We did not observe any significant difference in the proportion of CRF01_AE, CRF07_BC and subtype B among different ages between 2009 and 2013 (χ^2^ = 3.252, P = 0.354; χ^2^ = 7.263, P = 0.064; χ^2^ = 4.747, P = 0.191) (see Table 1). Several unique recombination forms (URF) among these study subjects were increasingly identified and confirmed by Bootscanning analysis (n = 24, 1.98%), which were either close to the CRF07_BC or CRF01_AE clusters and subtype B in phylogenetic trees (Fig 1). These URFs were CRF01_AE/BC (n = 14), CRF01_AE/B (n = 6), and CRF01_AE/C (n = 4), suggesting CRF01_AE strain plays a vital role in generating new recombinants. At least three different types of recombinants comprising of 01_AE/07_BC were found in three groups of patients, respectively (data not shown). The uniqueness in the clustering of these sequences suggests a new recombinant form which will be further analyzed, monitored, and reported elsewhere.

**Fig 2:**
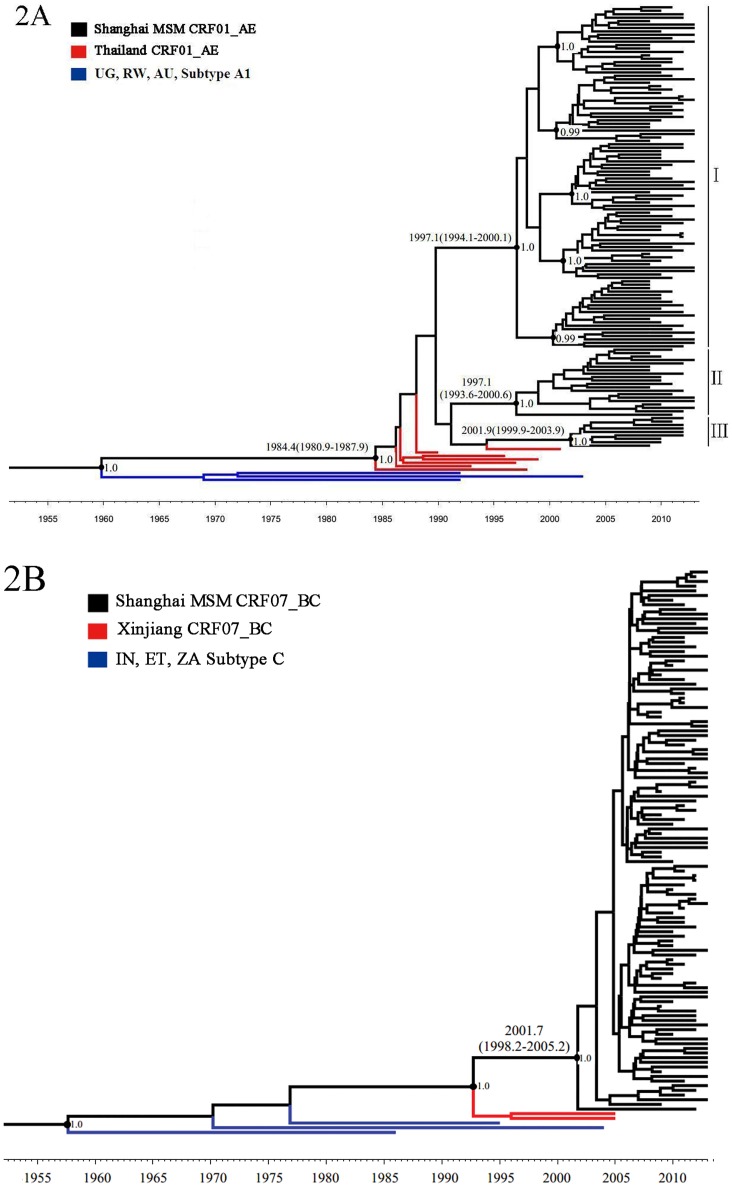
Maximum clade credibility (MCC) trees representing the rooted genealogy of CRF01_AE and CRF07_BC among MSM in Shanghai. 2A: the MCC tree for CRF01_AE strain. HIV-1 A1 sequences from Uganda(UG), Rwanda(RW), Australia(AU) and HIV-1 CRF01_AE sequences from Thailand (TH) were used as the references, including UG.92.AB253429, AU.03.DQ676872, RW.92.AB253421, TH.93.051, TH.90.U54771, TH.96.02138, TH.97.1695, TH.98.1251, TH.99.4460, and TH.01.2570. Blue lines represent A1 from UG, RW, AU and red lines represent CRF01_AE from TH. I, II, and III represent three clusters belonging to CRF01_AE strain. 2B: the MCC tree for CRF07_BC strain. HIV-1 subtype C sequence from Indian (IN), Ethiopia (ET), South Africa (ZA), and CRF07_BC sequence from Xinjiang, China (CNEF) were used as the reference, including IN.95.21068, ET.86.368370, ZA.04.AY772699, CNEF.05.368370, CNEF.05.368372. Blue lines represent subtype C from IN, ET, ZA and red lines represent CRF07_BC from CNEF. The branch lengths in the MCC tress reflect time and corresponding time-scale is shown at the bottom of the trees. Both the posterior probabilities and the tMRCA for the key nodes are indicated.

Bayesian inference analysis for the first time indicated that the transmission of CRF01_AE strain into MSM population was earlier than CRF07_BC strain in Shanghai based on tMRCA inference analysis. In MCC tree for CRF01_AE, three independent epidemic clusters (posterior = 1.0), one major (I), corresponding to CRF01_AE lineage 4 [25], and one small cluster (II), corresponding to CRF01_AE lineage 5 [25] and another small cluster (III), corresponding to Shanghai-based lineage, were identified, with different tMRCA at 1997.1 (1994.1–2000.1), 1997.1 (1993.6–2000.6), 2001.9 (1999.9–2003.9) respectively. The epidemic cluster I was expanding quickly and included most of studied sequences. Of interests, the epidemic cluster I consisted of five independent sub-clusters (posterior>0.95). However, only one cluster (posterior = 1.0) was found in MCC tree for CRF07_BC with a tMRCA at 2001.7 (1998.2–2005.2), whereas a potential tendency of two epidemic cluster-like existed. (Fig 2). The estimated evolutionary rates of both CRF01_AE and CRF07_BC at 2.11 (1.68–2.52)×10^-3^ and 1.56 (1.21–1.93)×10^-3^ substitutions/site per year, respectively, revealed on average, a faster genetic variation of CRF01_AE than that of CRF07_BC in these studied subjects. The genetic distance of CRF01_AE, CRF07_BC and B were 0.0379±0.0031, 0.0190±0.0016, 0.0640±0.0046 respectively, showed a difference significantly (P<0.001). Furthermore in pairwise comparison the genetic distance between CRF01_AE and CRF07_BC or subtype B was different significantly (P<0.05).

### Low level of baseline CD4+T cell count in CRF01_AE infections

CD4+T cell count is currently a major immunological indicator for classification of HIV-1 infection [26, 27] and initiation of antiretroviral treatment [28]. According to the US CDC classification for HIV/AIDS based on CD4+ T-Lymphocyte Categories [29], the CD4+T cell counts ≤200 cells/μl (category 1), 201–499 cells/μl (category 2), and ≥500 cells/μl (category 3), accounted for 13.30% (n = 168), 58.42% (n = 739), and 28.46% (n = 360), respectively in our study. Based on the US CDC classification for HIV/AIDS and our stratified analysis of CD4+T cells (see Table 2), 13.30% of them had been at the AIDS stage and almost half of the studied subjects had lower CD4+T cell level (n = 559, 44.2%, <350 cells/μl), which were definitely eligible for ARV therapy according to the currently available Chinese ART protocol [28].

**Table 2:** The stratified baseline CD4+T cells and viral loads based on HIV-1 different subtypes.

	HIV-1 Subtypes [n(%)]				Total	χ^2^	*P* value	
	CRF01_AE	CRF07_BC	Subtype B	Others*				
**CD4^+^ cell counts**	n = 786(62.1)	n = 310(24.5)	n = 102(8.1)	n = 67(5.3)	n = 1265(100)	26.175	0.002
(cells/μl) ≤200	129(16.4)	19(6.1)	15(14.7)	5(7.5)	168(13.3)			
201–350	242(30.8)	98(31.6)	27(26.5)	24(35.8)	391(30.9)		
351–499	206(26.2)	91(29.4)	29(28.4)	22(32.8)	348(27.5)		
≥500	209(26.6)	102(32.9)	31(30.4)	16(23.9)	358(28.3)		
**Viral loads** **(copies/ml)	n = 180(59.8)	n = 77(25.6)	n = 25(8.3)	n = 19(6.3)	n = 301(100)	8.578	0.172
<10000	13(7.2)	8(10.4)	2(8.0)	0(0.0)	23(7.6)		
10000–100000	102(56.7)	50(64.9)	14(56.0)	8(42.1)	174(57.8)		
>100000	65(36.1)	19(24.7)	9(36.0)	11(57.9)	104(34.6)		

* HIV-1 CRF55_01B, CRF59_01B, CRF67_01B, CRF68_01B and URFs.

** Viral loads from 301 HIV-1-infected patients.

Age-dependent analysis indicated that baseline CD4+T cell counts was significantly different amongst four aged groups (H = 38.652, P<0.001). The persons who aged ≤ 25 years, 26–35 years, 36–45 years, and ≥ 45 years had CD4+T cell counts with 418 (297.5–542 cells/μl), 385.5 (270.5–523.75 cells/μl), 352.5 (239–467.75 cells/μl), and 286.9 (174–436 cells/μl), respectively, whereas, significant difference in CD4+T cell counts was only present amongst subjects ≤25 and ≥45 years old (H = 230.907, P<0.001). (see S1 Table.)

**Fig 3:**
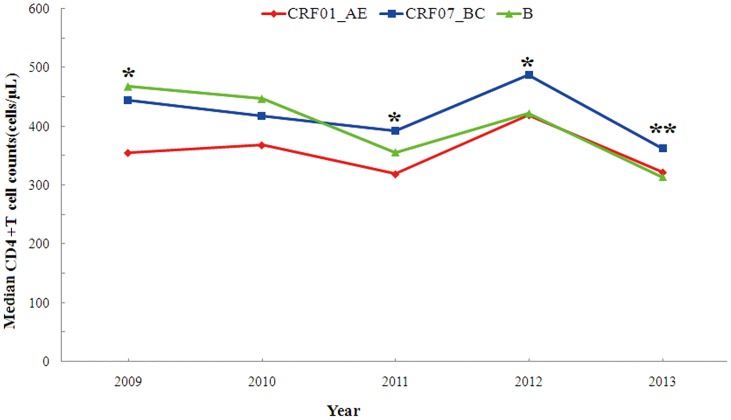
Association of three different subtype strains circulating among HIV-1-infected MSM from 2009 to 2013 with baseline CD4+T cell counts. *: P<0.05 when comparing CRF01_AE with CRF07_BC; **: P<0.01 when comparing CRF01_AE with CRF07_BC.

Overall, the proportion of CD4+T cell counts in studied subjects infected by different HIV-1 subtype strains showed a significant difference (P<0.01, see Table 2). Of particular importance was that CD4+T cells in CRF01_AE infection was significantly lower than that in CRF07_BC infection during 5 years (Fig 3), similar to our previously small-scale study focusing on persons of age <25 years old [17]. The median baseline CD4+T cell count on five-year showed different levels with 366 (255.5–503.4 cells/μl), 415 (304–553.6 cells/μl), 376 (256–557 cells/μl) for CRF01_AE, CRF07_BC, subtype B, respectively, (H = 17.919, P<0.001). Further analysis with a pairwise comparison indicated a significant difference in CD4+T cells between CRF01_AE and CRF07_BC (H = -97.326, P<0.001), but not shown between subtype B and CRF01_AE or CRF07_BC (H = -34.114, P = 0.342; H = 63.212, P = 0.315) (Fig 3).

### High level of baseline viral load in CRF01_AE infection

**Fig 4:**
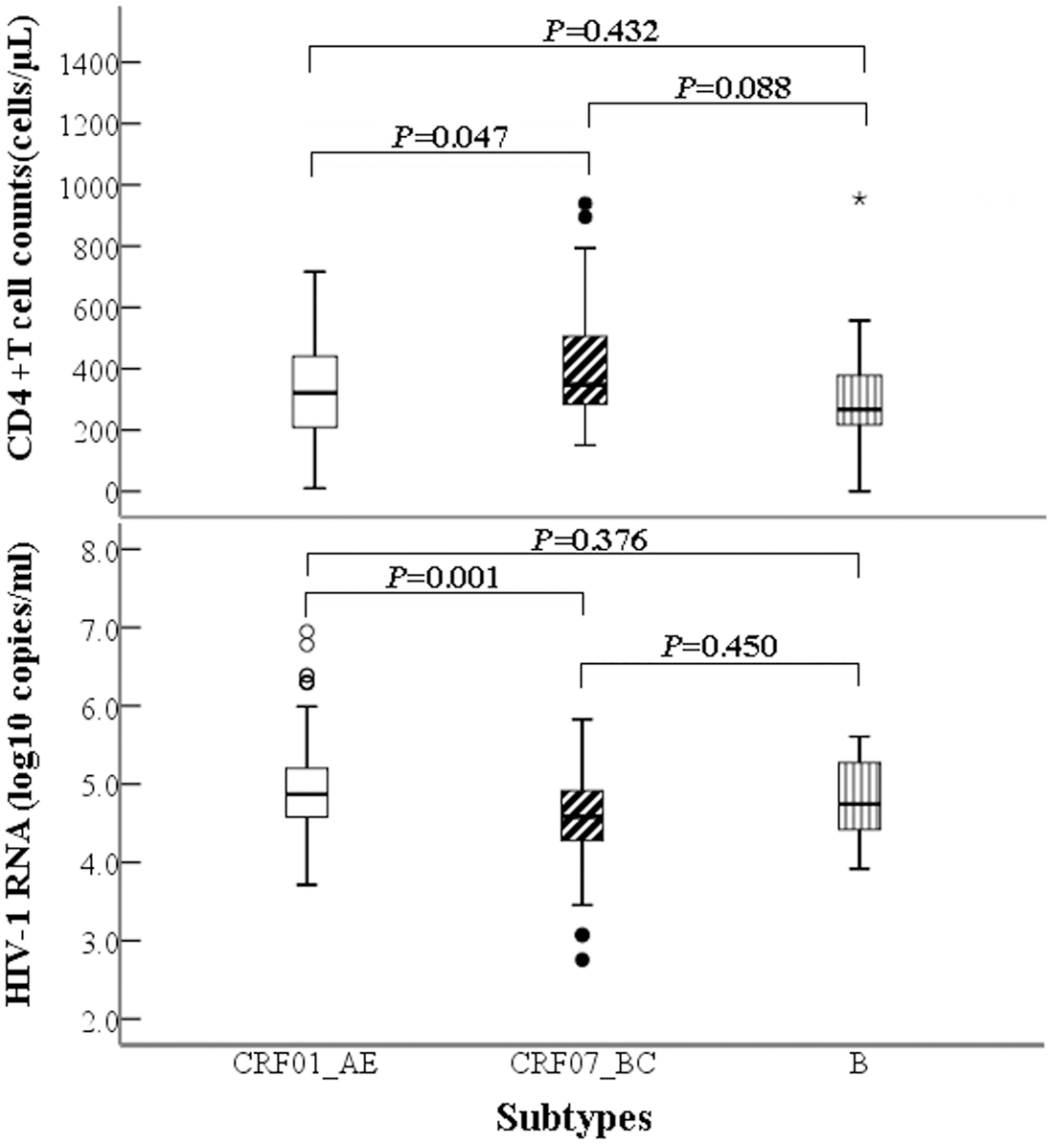
Comparisons of median baseline CD4+T cell counts and viral loads between three different subtypes. Median baseline CD4+T cell was lower in CRF01_AE infection than in CRF07_BC infection (P<0.05) while median baseline viral load was higher in CRF01_AE infection than in CRF07_BC infection (P = 0.001).

Giota Tooloumi’s et al [21] reported that HIV-1 subtype in European seroconverter cohorts significantly influences seroconversion CD4 cell levels and decline rates but not viral load set point. We compared the different subtype strains on the basis of the corresponding baseline viral load, which was measured at the same time as CD4+ T cell counting, among 301 patients’ samples derived from year 2013. Overall, the viral loads were 4.87 (4.57–5.21 log10 copies/ml), 4.58 (4.27–4.93 log10 copies/ml), and 4.74 (4.41–5.27 log10 copies/ml) for CRF01_AE, CRF07_BC and subtype B, respectively, showing a significant difference (H = 14.194, P = 0.001) (see S1 Table and Fig 4). For the 301 samples analyzed, CD4+T cell count was lower in CRF01_AE than CRF07_BC infected patients, however, on the contrary, viral load was higher in CRF01_AE than in CRF07_BC infected patients (H = 41.245, P = 0.001), but this difference was not seen between CRF01_AE and subtype B patients (H = 14.949, P = 0.376). Of interests is that the viral load varied in different age groups. Viral loads of 4.74 (4.25–5.07), 4.81 (4.46–5.18), 4.79 (4.50–5.12), and 4.99 (4.82–5.41) log10 copies/ml were for age group ≤25, 26–35, 36–45, ≥45 years old, respectively. However, the only viral load for persons aged ≥45 years was significantly higher than those aged ≤25 years (H = -54.848 P = 0.024), similar to previous reports [21, 30].

## Discussion

Shanghai, a metropolis of economic prosperity, well-developed transportation and communication, and multi-culture, attracts increasing numbers of migrants. By the end of 2013, more than 24 million people are permanent residents including 9.9 million migrant resident populations. In addition, well-improved sense of stigma and discrimination for MSM made them flock into metropolises or big cities, where a closely larger social and sexual network more or less had been established or could be easily constructed. It is worthwhile to note that MSM are currently vulnerable to HIV infection in Shanghai, where roughly 80,000 MSM individuals gather. Different epidemiological studies worldwide had revealed some risk factors for HIV acquisition in MSM at individual levels, including unprotected anal intercourse, high frequency of sexual activity with multiple male sex partners, and high prevalence of sexually transmitted diseases (STD) among this population [31, 32]. Therefore, the continuous migration waves for years have unavoidably brought a high prevalence of HIV-1-infection in Shanghai [17].

Our phylogenetic analysis based on *pol* gene clearly revealed the presence of viral subtype diversity among the studied subjects covering all ages. Obviously, the major HIV-1 subtype is still CRF01_AE, reaching 62.13% in 2013, compared to 42.9% in 2005 [33], followed by 24.43% for CRF07_BC, the second dominant strain. However in the recent 5 years, the proportion of CRF01_AE showed a tendency of decreasing (P-trend = 0.003). One possible explanation for this phenomenon is that the proportion of the second-generation recombination of CRF01_AE with other subtype and/or recombinant has been increasing. Overall, in our study, the change of proportion of CRF01_AE plus other recombinants related to CRF01_AE was not of significant difference in recent 5 years (χ^2^ = 8.579, P = 0.073; Trend: χ^2^ = 1.134, P-trend = 0.287). However, the change of proportion of new recombinants like CRF55_01B together with other undefined recombinants related to CRF01_AE seems to be of significant difference (χ^2^ = 15.805, P = 0.03; Trend: χ^2^ = 3.440, P-trend = 0.064). In this study, we also determined at least 42 recently identified CRFs and 24 URFs as well, indicating the viral genetic heterogeneity and subtype/recombinant complexity among MSM epidemic in this city. It is well known that CRF01_AE caused an outbreak among the high-risk heterosexual population in Thailand in the late 1980s [34, 35], and was subsequently disseminated to various risk populations in neighboring countries, including China [6, 36, 37]. Of note, many of CRF01_AE-based inter-subtype or inter-recombinants such as CRF55_01B, CRF59_01B, CRF67_01B, CRF68_01B, and some URFs [12, 15, 16, 38] have been identified recently in different provinces in China, suggesting the future epidemic will even be broader. Based on our current finding that not only did CRF55_01B, originated in Shenzhen city [16] and firstly identified in our samples in 2011, have an increasing trend with years, but also different mosaic forms of 01AE/07BC recombination were being transmitted in Shanghai. These new type recombinant strains could be an alert for a future epidemic. By analysis of pairwise genetic distance among three subtypes, CRF01_AE, CRF07_BC and subtype B, a higher genetic diversity of AE and B than 07BC was observed, indicating a possible mechanism with either multiple introductions of these two strains into the MSM population or a longer circulating time of CRF01_AE and subtype B than of CRF07_BC (*P*<0.01).

Analysis of tMRCA using molecular clock principle can be used to estimate when the viral epidemic began and to estimate the early growth rate [39]. We found that tMRCA of CRF01_AE among MSM population in Shanghai were in 1997 (cluster I and II) and in 2001 (cluster III), similar to other previous study [25], in which tMRCA of CRF01_AE among MSM in China was estimated from mid to late 1990s. In addition, we found that the tMRCA of CRF01_AE cluster I and II was earlier than CRF07_BC (2001) other than CRF01_AE cluster III (2001), indicating an earlier introduction of CRF01_AE strain than CRF07_BC strain into MSM in Shanghai. Unexpectedly, one unique Shanghai-based epidemic cluster (III) and five independent sub-clusters contained within major cluster (I) in MCC tree for CRF01_AE were observed (Fig 2), implying that a divergent evolution of CRF01_AE strain has occurred, and five independent transmission networks might have been established after entering MSM population in this city. In this study, we for the first time determined the tMRCA of both CRF01_AE and CRF07_BC strains, and identified multiple epidemic sub-clusters of CRF01_AE strain circulating among MSM population in Shanghai. However, these epidemic clusters and/or sub-clusters in relation to their origin of virus introduction, social and sexual network, as well as cross-transmission between different high-risk groups would deserve further investigation in our future study.

HIV infection in ART-naive individuals is typically characterized by a decline in CD4+T cell count and a rise in plasma HIV RNA (viral load), which eventually leads to opportunistic infections, development of AIDS and AIDS-related deaths if left untreated. Therefore, early detection of HIV infection, timely monitoring of disease progression, and early linkage to ART are undoubtedly critical steps to curbing the spread of HIV. Patients who meet the treatment criteria of CD4+T cell count ≤200 cell**s/**μl (revised to ≤350 cells/μl in 2008, and ≤500 cells/μl in 2014) are eligible for ART according to National Free Antiretroviral Therapy Program (NFATP) [28]. A dramatic reduction in mortality among patients receiving ARV treatment over recent years greatly benefited from NFATP [40]. Therefore, after the diagnosis of HIV infection, timely CD4+T cell counting is a crucial step in determining whether the patients meet the criteria for ART initiation. This 5-year retrospective analysis showed that the baseline CD4+T cells count**s** at the first follow-up were tested in 1 month on average (0.97; 0.53–1.57 month) after HIV-1 diagnosis. It was important to note that baseline CD4+T cell count ≤200 cells/μl and ≤350 cells/μl accounted for 13.3% and 44.2%, respectively in our study, compared to Wu’s report [41] in which 18.77% and 45.06% were found among newly diagnosed MSM. The difference in baseline CD4+T cell level between the two studies (≤200 cells/μl: χ^2^ = 24.337, *P*<0.01) could be the different sample inclusion criteria, sampling scale, and HIV-1 subtypes as well.

HIV-1 infection may have different impact on disease progression among infected individuals [19, 21] since its extensive genetic heterogeneity [42, 43]. Several noticeable findings [20, 21] drew our attention. A statistical significant four-fold faster of CD4+T cell decline and a higher rate of virological rebound in subtype D infections was found compared to other subtype infections [20]. Besides, lower CD4+T cell count in subtype C infections and higher in CRF01_AE infections at seroconversion was discovered and thenCD4+T cell loss in CRF01_AE and subtype B was faster than subtype C at 2 and 4 years after seroconversion although no significant change in viral load set point was observed in subtype C infections [21]. Our previous study though with a small sample size (n = 364) of patients of age ≤ 25 years old indicated that the CRF01_AE variant could be more pathogenic than other subtypes and/or recombinant stains [17]. Lower baseline CD4+T cell counts and fast depletion in patients could be due to the trait of CXCR4 co-receptor usage of CRF01_AE strains [17, 18]. However, information on the impact of HIV-1 subtypes on baseline CD4+T cells, viral load and disease progression in large-scale samples remains rather sparse in China [17, 18]. Our present study found a lower level of baseline CD4+T cell count among patients infected with CRF01_AE strain than those infected with CRF07_BC in 1265 individuals’ samples, strongly suggesting that the pathogenecity of CRF01_AE strain might play a significant role in early CD4+T cells depletion and possible fast disease progression. Nevertheless, the subtype- and recombinant-associated biological and immunological mechanisms remain to be further elucidated.

Our analyses of 301 samples from the studied subjects who received a baseline detection of viral load showed that median viral load was higher in CRF01_AE infection than in CRF07_BC infection (p<0.01), indirectly supporting the finding of an increased rate of CD4+T cell count decline in CRF01_AE-infected patients compared with non-CRF01_AE patients [44]. We observed that lower CD4+T cell count was associated with higher viral load, with a significant difference between viral load <10000 and >100,000 copies/ml (P = 0.018), and between viral load 10,001–100,000 and >100,000 copies/ml (P = 0.001) based on the median CD4+T cell counts, which is consistent with the Martinson et al report [45].

Similar to other reports [21, 30, 41], we likewise observed a lower baseline CD4+T cell count in older (over 45 years) than in younger individuals (under 25 years) (p<0.05) and a higher baseline viral load in older than in younger individuals (p<0.05), indicating the need for early medical care and intervention among HIV infected people >45 years of age.

In conclusion, this study illustrated HIV-1 phylogenetic diversity among MSM in Shanghai, and further demonstrated a stronger pathogenetic virulence of CRF01_AE strain compared to CRF07_BC by analysis of baseline CD4+T cell and viral load. Determination of tMRCA of CRF01_AE and CRF07_BC would help us get a better understanding of different viral epidemics locally. Further study is still needed to elucidate the correlation between phylogenetic clusters and socio- and sex network among MSM population that may help us effectively implement directional intervention.

## Supporting Information

S1 Table Age-associated median baseline CD4+T cell counts and viral loads based on three different HIV-1subtypes. (DOC)
