# Supplementary material for: HIV-1 Genetic Diversity and Its Impact on Baseline CD4+T Cells and Viral Loads among Recently Infected Men Who Have Sex with Men in Shanghai, China
Source: PLoS One. 2015 Jun 29;10(6):e0129559. doi: 10.1371/journal.pone.0129559 (PMC4486722; doi:10.1371/journal.pone.0129559)
Supplement: S1 Table — (DOC) [file pone.0129559.s001.doc]

|  | **HIV subtype** | | | |  |  |
| --- | --- | --- | --- | --- | --- | --- |
|  | **CRF01_AE** | **CRF07_BC** | | **B** | ***P* value** | |
| **CD4 cell counts(cells/μl)**  Total | 366(255-503)* | 415(304-553) | 376(256-557) | | <0.001 |  |
| Age |  |  |  | |  |  |
| ≤ 25 years | 410(279-525) | 425(321-573) | 452(357-583) | | 0.065 |  |
| 26-35 years* | 369 (260-512) | 415(298-551) | 358(259-556) | | 0.043 |  |
| 36-45 years | 336(239-451) | 426(258-498) | 344(186-581) | | 0.209 |  |
| ≥ 46 years | 269 (131-417) | 343 (216-512) | 235(143-357) | | 0.098 |  |
| **Viral Load(log10 copies/ml)****  Total | 4.87 (4.57-5.21)* | 4.58 (4.27-4.92) | 4.74 (4.41-5.27) | | 0.001 |  |
| Age |  |  |  | |  |  |
| ≤ 25 years | 4.83(4.34-5.17) | 4.56(4.18-4.92) | 4.75(4.14-5.19) | | 0.420 |  |
| 26-35 years | 4.83(4.57-5.18) | 4.60(4.28-5.04) | 4.73(4.52-5.25) | | 0.154 |  |
| 36-45 years | 4.79(4.56-5.06) | 4.61(4.30-4.61) | 4.74(4.33-5.48) | | 0.893 |  |
| ≥ 46 years | 5.01(4.88-5.58) | 4.89(4.78-5.05) | 4.93(4.35-5.41) | | 0.416 |  |

**S1 Table. Age-associated median baseline CD4+T cell counts and viral loads based on three different HIV-1subtypes**

* P <0.05 when comparing between CRF01_AE and CRF07_BC

** Viral loads from 301 HIV-1-infected patients.
